# Suppressed rate of carcinogenesis and decreases in tumour volume and lung metastasis in CXCL14/BRAK transgenic mice

**DOI:** 10.1038/srep09083

**Published:** 2015-03-13

**Authors:** Ryu-Ichiro Hata, Kazuhito Izukuri, Yasumasa Kato, Soichiro Sasaki, Naofumi Mukaida, Yojiro Maehata, Chihiro Miyamoto, Tetsu Akasaka, Xiaoyan Yang, Yoji Nagashima, Kazuyoshi Takeda, Tohru Kiyono, Masaru Taniguchi

**Affiliations:** 1 Oral Health Science Research Center, Graduate School of Kanagawa Dental University, Yokosuka. 238-8580, Japan; 2 Department of Oral Science, Graduate School of Kanagawa Dental University, Yokosuka. 238-8580, Japan; 3 Department of Oral Function and Molecular Biology, Ohu University School of Dentistry, Koriyama. 963-8611, Japan; 4 Division of Molecular Bioregulation, Cancer Research Institute, Kanazawa University, Kanazawa. 920-1192, Japan; 5 Department of Molecular Pathology, Yokohama City University Graduate School of Medicine, Yokohama, 236-0004, and Department of Surgical Pathology, Tokyo Women's Medical University Hospital, Tokyo. 162-8666, Japan; 6 Department of Immunology, Juntendo University School of Medicine, Tokyo. 113-8421, Japan; 7 Division of Carcinogenesis and Cancer Prevention, National Cancer Center Research Institute, Tokyo. 104-0045, Japan; 8 Laboratory for Immune Regulation, RIKEN Center for Integrative Medical Sciences, Yokohama. 230-0045, Japan

## Abstract

Cancer progression involves carcinogenesis, an increase in tumour size, and metastasis. Here, we investigated the effect of overexpressed CXC chemokine ligand 14 (CXCL14) on these processes by using CXCL14/BRAK (CXCL14) transgenic (Tg) mice. The rate of AOM/DSS-induced colorectal carcinogenesis in these mice was significantly lower compared with that for isogenic wild type C57BL/6 (Wt) mice. When tumour cells were injected into these mice, the size of the tumours that developed and the number of metastatic nodules in the lungs of the animals were always significantly lower in the Tg mice than in the Wt ones. Injection of anti-asialo-GM1 antibodies to the mice before and after injection of tumour cells attenuated the suppressing effects of CXCL14 on the tumor growth and metastasis, suggesting that NK cell activity played an important role during CXCL14-mediated suppression of tumour growth and metastasis. The importance of NK cells on the metastasis was also supported when CXCL14 was expressed in B16 melanoma cells. Further, the survival rates after tumour cell injection were significantly increased for the Tg mice. As these Tg mice showed no obvious abnormality, we propose that CXCL14 to be a promising molecular target for cancer suppression/prevention.

Side effects are the most serious obstacles in the case of cancer therapeutics^1^,^2^,^3^,^4^. Thus, prevention of cancer remains the most promising strategy for reducing its incidence and associated mortality due to this disease^5^,^6^. Tumour progression has been shown to be largely dependent on the expression of tumour-promoting and tumour-suppressing genes, with the balance being in favour of the former at each step^7^. The protein products of these oncogenes and tumour suppressor genes function as regulatory intracellular signalling molecules during this process. Recently, it was revealed that the cancer microenvironment also influences carcinogenesis and cancer progression^8^,^9^.

In our previous search to find endogenous tumour suppressors functioning to prevent head and neck squamous cell carcinoma (HNSCC), we cultured HNSCC cells under serum-free conditions and treated them with epidermal growth factor, whose receptor is frequently hyperactive in HNSCC and cancers of other tissues, and focused on molecules down regulated in this type of cancer. In that study, CXC chemokine ligand 14 (CXCLl4), also known as breast and kidney expressed chemokine (BRAK), was found to be significantly down regulated^10^. Interestingly, the expression of CXCL14 was also shown to be down regulated in tissues obtained from patients with HNSCC^11^.

Chemokines (chemotactic cytokines) are a group of structurally related proteins with molecular weights in the range of 8 k to 12 k that have been reported to regulate the cellular trafficking of various types of leukocytes by interacting with a subset of G protein-coupled receptors^12^. Each chemokine is named according to the arrangement of the cysteine residues within it. Further, the two major subfamilies, defined by the presence of four conserved cysteine residues linked by two disulphide bonds, are the CC and CXC chemokines. They are distinguished according to the position of the first two-cysteine residues, which are adjacent to each other (CC subfamily) or separated by one amino acid (CXC subfamily). In the tumour microenvironment, chemokine expression acts to determine the distribution of immune cells, and it thus controls the overall immune response to the tumour, and plays an integral role in the regulation of cancer progression and metastasis^13^,^14^,^15^,^16^.

CXCL14 is a non-ELR (Glu–Leu–Arg) CXC chemokine and is expressed ubiquitously and constitutively in epithelia throughout the body, and several physiological functions of it have been proposed, such as recruitment and maturation of monocyte-derived macrophage and renewal of Langerhans cells in the skin. Promotion of trafficking of matured natural killer cells to the sites of inflammation and macrophage infiltration into white adipose tissue in obese mice fed a high-fat diet, as well as inhibition of angiogenesis, were also reported as functions of this chemokine^17^.

In order to further investigate whether CXCL14 has a tumour-suppressing effect *in vivo*, we prepared and cloned CXCL14-expression vector-transfected and mock vector-transfected tongue tumour-derived cells. The rate of tumour formation in athymic nude mice or in severe combined immunodeficiency (SCID) mice following xenotransplantation was significantly lower for the CXCL14-expressing cells than for the mock-transfected cells, even though no differences were observed in the growth rates of these cells under *in vitro* culture conditions^18^,^19^. These data indicate that CXCL14 expression in tumour cells functioned to suppress the growth of these cells *in vivo*^18^,^19^,^20^. Next, in order to confirm whether CXCL14 would have a tumor suppressing effect on cells of other tissue origins, we produced transgenic (Tg) mice expressing 10-fold higher blood CXCL14 compared with the level produced by isogenic wild-type C57BL/6 (Wt) mice and found that these Tg mice significantly suppressed increase in the size of tumours formed by transplanted B16 melanoma cells or Lewis lung carcinoma (LLC) cells compared with Wt mice^17^,^21^.

The multistep nature of tumour formation has been well established and each step depends on the mutation or abnormal regulation of various genes^22^,^23^,^24^. In order to elucidate the *in vivo* function of CXCL14, in this present study we used CXCL14 transgenic (Tg) mice and investigated the effects of this chemokine at multiple stages during cancer development, including carcinogenesis, increase in tumour size, and tumour metastasis, in addition to the effects on the overall survival rate. Furthermore, we also sought to determine the role of CXCL14 on the functions of natural killer (NK) and natural killer T (NKT) cells.

## Results

### Rate of chronic colitis-associated carcinogenesis was suppressed in CXCL14 Tg mice

**Figure 1:**
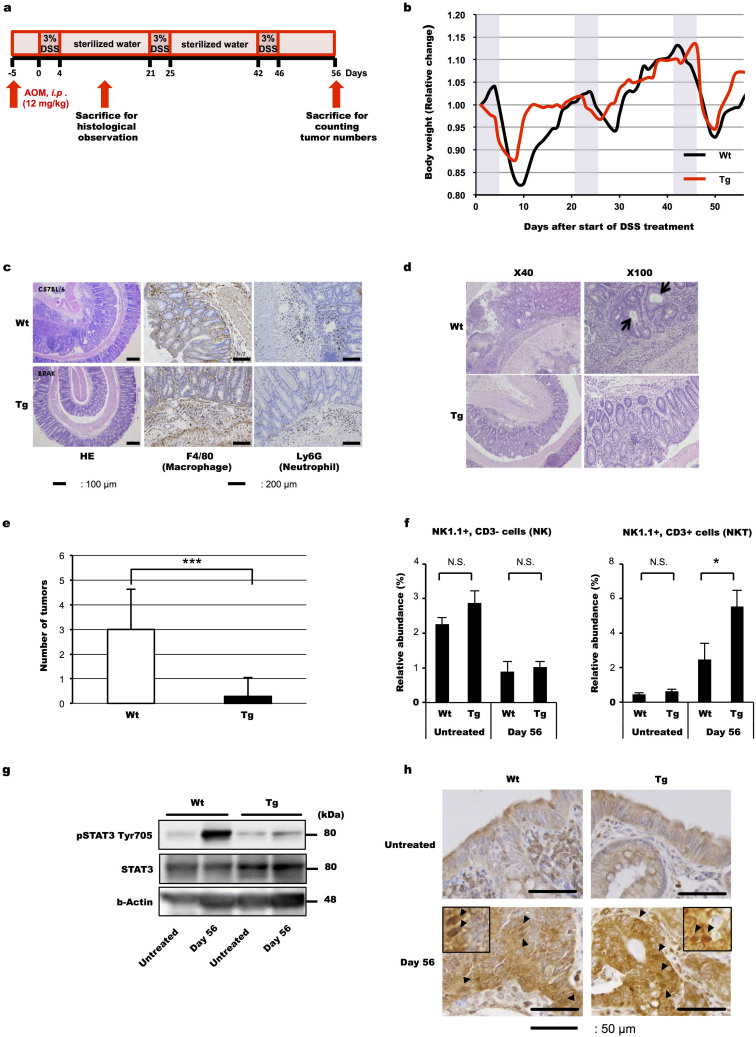
Suppressed chronic colitis-associated carcinogenesis in CXCL14 transgenic (Tg) mice. (a) Experimental design utilized to promote inflammation-driven colonic tumorigenesis. AOM; azoxymethane, DSS; dextran sodium sulphate. (b) Effect on body weight changes in Wt and Tg mice treated with DSS after AOM injection. Representative results from 5 mice are shown here. Body-weight changes calculated as the relative change from the body weight at the start of DSS treatment. (c, d) Representative images (n = 5) for the histological analysis of colon tissue isolated from wild type (Wt) and CXCL14 transgenic (Tg) mice at 14 days (c) and 56 days (d) after the initial intake of DSS. The colon tissue samples obtained at 14 days were processed for both haematoxylin and eosin (HE) staining and immunohistochemical analysis using anti-F4/80 and anti-Ly6G antibodies to detect infiltrating macrophages and neutrophils, respectively; whereas the samples obtained at 56 days were stained with hematoxylin and eosin only. In panel “c”, the thin and bold scale bars indicate 100 μm and 200 μm, respectively. Arrows in “d” indicate fused glands with enlarged hyperchromatic nuclei. (e) Reduced tumour incidence in CXCL14 Tg mice compared with that in Wt mice. The colon tissues were obtained and the tumours were counted macroscopically (n = 8). (f) Single cell suspensions were prepared from colon tissues of Wt or Tg mice on day 0 or day 56. The resultant cells were analyzed on a FACSCanto System II as described under Materials and Methods (n = 5). (g) Colon tissues were obtained as in “f” and proteins were separated as described under Materials and Methods, transferred onto nylon membranes, and then reacted with antibodies against STAT3 and pSTAT3. Beta actin was employed as an internal standard. Representative results from 5 independent experiments are shown here. (h) The sections were obtained before (Untreated) and after AOM/DSS treatment (Day 56) and reacted with anti-NF-κB p65 antibody. The insets show enlarged intranuclear staining. Representative results from 5 independent experiments are shown here. Scale bars represent 50 μm. Data are the means ± S.D. * *P* < 0.05, *** *P* < 0.001, Student's *t*-test.

The protocol utilized to promote inflammation-driven colonic tumourigenesis, azoxymethane (AOM)/dextran sodium sulphate (DSS)-induced cancer, is illustrated in Fig. 1a. Supplementation of the drinking water with DSS similarly down-regulated the body weight of both Wt and Tg mice (Fig. 1b). Haematoxylin and eosin (HE)-staining and immunohistochemical analysis of the colon sections at 14 day after the initial ingestion of DSS revealed the presence of more pronounced inflammatory infiltrates, which included macrophages and neutrophils, in the wild type (Wt) mice than in the Tg mice (Fig. 1c). Sections obtained from the distal colon taken at 56 days showed an obvious decrease in the number of carcinogenic foci, which were composed of fused glands with enlarged hyperchromatic nuclei, in the Tg compared with that in the Wt (Fig. 1d). The incidence of AOM/DSS-induced cancer in the CXCL14 Tg mice was significantly lower (*P* < 0.001) than that observed for the Wt ones (Fig. 1e). In order to investigate the effects of CXCL14 on NK cells and NKT cells and metabolism, we performed additional experiments and found that relative number of NK (NK1.1^+^, CD3^−^) cells was not different between Wt and Tg mice either before or after treatment with AOM/DSS. On the other hand, that of NKT (NK1.1^+^, CD3^+^) cells was significantly increased after AOM/DSS treatment (Fig. 1f). Also a significant increase in activated STAT3 (phospho-STAT3 Tyr705) was observed after treatment with AOM/DSS in the Wt mice but not in the Tg ones after treatment with AOM/DSS (Fig. 1g). Positive staining of intranuclear p65 subunit of nuclear factor κB (NFκBp65) was observed both in Wt and Tg mice tissue only after treatment with AOM/DSS (Fig. 1h).

### NK cell depletion attenuated the suppressive effects of CXCL14 on the increase in tumour volume

**Figure 2:**
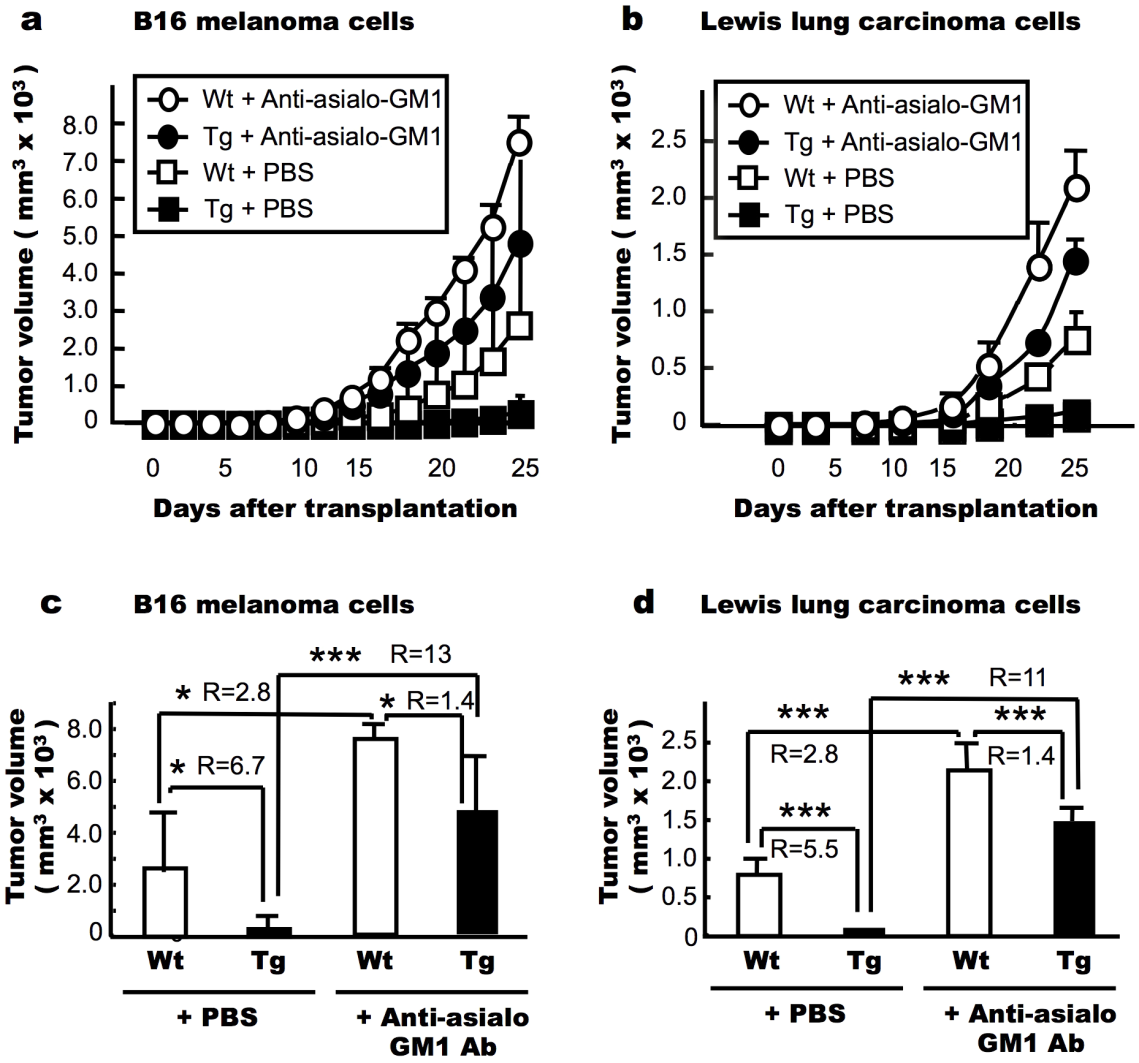
Anti-asialo-GM1 antibody attenuates the growth-suppressive effects of tumour cell transplants in CXCL14 transgenic (Tg) mice. B16 melanoma cells (a, c) or Lewis lung carcinoma cells (LLC; b, d) were inoculated (1 × 10^5^ cells/site) into both sides of the dorso-lateral region of 8 (B16) or 10 (LLC) female Wt and Tg mice (homozygous line 20). Half of these mice were injected with anti-asialo-GM1 antibody (0.5 mg/200 μL/animal) 3 days before tumour cell inoculation and once a week thereafter to deplete NK cells. The final volume of the tumours at day 25 is indicated in panels “c” and “d”. R indicates the tumour size ratio between the two groups connected with a bracket. Data are means ± S.D. * *P* < 0.05, *** *P* < 0.001, Student's *t*-test.

B16 melanoma cells or Lewis lung carcinoma (LLC) cells were inoculated into both sides of the dorso-lateral region of female Wt and Tg mice. Significant differences in tumour volume were observed between the Wt and Tg mice for both the B16 melanoma (Fig. 2a and c (+PBS), *P* < 0.05) and LLC (Fig. 2b and d (+PBS), *P* < 0.001) cells at day 25 after transplantation. Three intraperitoneal (*i.p.*) injections of anti-asialo-GM1 antibody, which depletes NK cells, further increased the volume of tumour cell transplants in the Wt mice 2.8-fold for both the melanoma (R in Fig. 2c) and LLC (R in Fig. 2d) cells. The injection of this antibody also attenuated the suppressive effects of CXCL14 on tumour volume in the Tg mice, resulting in more pronounced increases of 13- and 11-fold for the melanoma and LLC cells, respectively (R in Fig. 2c and d). Thus, the ratio of Wt to Tg tumour size was decreased from 6.7 to 1.4 for the B16 melanoma cell transplants (Fig. 2c) and from 5.5 to 1.4 for the LLC cell transplants (Fig. 2d) following the injection of the anti-asialo-GM1 antibody. These data indicate that NK cell activity played an important role during the suppression of the increase in tumour volume in both Wt and Tg mice, and that the participation of NK cells in this process was enhanced when CXCL14 was overexpressed *in vivo*.

### Effects of anti-asialo-GM1 and anti-NK1.1 antibodies on the metastatic rates of tumour cells

**Figure 3:**
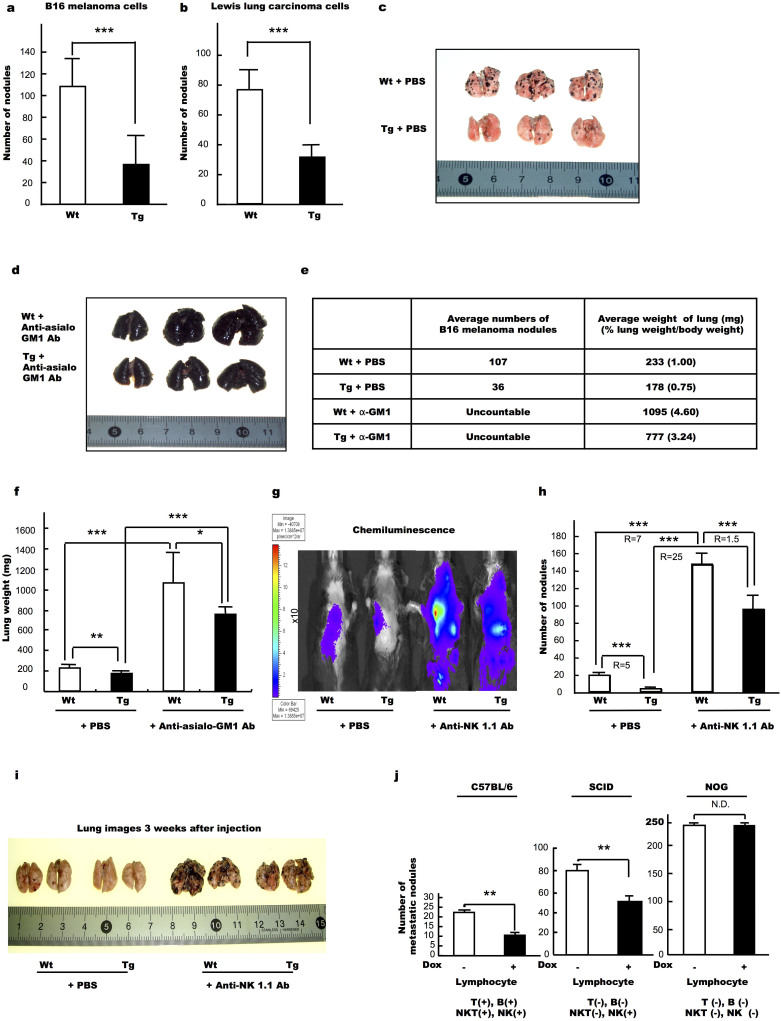
NK cell-dependent suppression of tumour cell metastasis in Wt and CXCL14 transgenic (Tg) mice. (a, b) B16 melanoma cells (a) or Lewis lung carcinoma (LLC) cells (b) were injected (2 × 10^5^ cells) into a tail vein of 12 each (B16) or 18 each (LLC) of the Wt and CXCL14 Tg mice. After 18 days, the metastatic nodules in the lungs were counted. (c, e and f) B16 melanoma cells were injected (2 × 10^5^ cells) into a tail vein of 14 Wt and 17 Tg mice. About half of these mice were injected with anti-asialo-GM1 antibody (αGM1, 0.5 mg/200 μL PBS/animal) 3 days before melanoma cell inoculation and thrice a week thereafter to deplete NK cells. Lung images of the PBS injected animals (c) and the anti-asialo-GM1 antibody injected animals (d) are shown. The number of tumour cell metastases and/or lung weights are also given (e) and compared (f). (g–i) Effects of the anti-NK1.1 antibody on the metastasis of B16-luc2LMT3 cells in Wt and Tg mice were determined by injecting the melanoma cells (2 × 10^5^ cells/200 μL PBS), expressing the luciferase reporter gene, into a tail vein of each of 12 Wt and Tg mice. Half of these mice were injected with an anti-NK1.1 antibody (50 μg/50 μL PBS/animal) 3 days before inoculation and then once a week thereafter to deplete NK and NKT cells. *In vivo* luciferase activity of the injected B16-luc2 LMT-3 was determined by measuring the chemiluminescence 30 minutes after luciferin injection by using an IVIS 50 (g). The number of metastatic nodules observed in the lungs 3 weeks after the injection of the cells (h) and lung images 3 weeks after injection of the cells (i) are shown. (j) B16-luc2Tet/OnBRAK melanoma cells were injected via a tail vein of Wt, SCID and NOG mice and the animals were fed 5% sucrose solution with or without 0.2% doxycycline (Dox). R indicates the ratio of nodule numbers between Wt and Tg mice or mice treated and not treated with the antibody. Data are means ± S.D. ** *P* < 0.01, *** *P* < 0.001, Student's *t*-test.

In addition to the changes in tumour volume, we also found that lung metastasis of LLC cells was lower in Tg mice than in the Wt ones. However, the lower extent of metastasis in the Tg mice could have been due to a small number of tumour cells in the original tumours and not due to suppression of metastasis in the Tg mice. In order to investigate the actual effect on metastasis in Tg mice, we employed an experimental metastasis system. To do this, we injected B16 melanoma or LLC cells into the tail veins of Wt and Tg mice and then counted the number of metastatic nodules in the lungs at day 18 post-injection (see also Supplementary Figure S1). For both melanoma cells (Fig. 3a) and LLC cells (Fig. 3b), the number of nodules in the lungs of the Tg mice was significantly (*P* < 0.001) lower than that observed for the Wt animals. In this analysis, half of the Wt and CXCL14 Tg mice were injected (*i.p.*) with anti-asialo-GM1 antibody in order to investigate the participation of NK cells in the suppression of tumour cell metastasis. Before injection, a clear visible difference in the degree of metastasis was observed between B16 melanoma cell injected Wt and Tg mice (Fig. 3c), with the number of B16 melanoma nodules being significantly lower in the Tg group (Fig. 3e). The numbers of metastatic nodules in Tg and their Wt mice were 36 and 107, respectively, so that the rate of pulmonary metastasis in Tg mice was one third of that of their Wt counterpart. When the mice were injected with anti-asialo-GM1 antibody in order to deplete NK cells, the lungs obtained from both Wt and Tg mice were completely filled with melanoma cells (Fig. 3d) such that the number of nodules could not be counted. Notably, the lung weights were significantly increased following injection of the antibodies (Fig. 3 e–i), and the percent lung weight per body weight was likewise increased in both Wt and Tg mice (Fig. 3e).

To further confirm the participation of NK cells and NKT cells in the process of tumour metastasis, anti-NK 1.1 antibody was also injected into the mice before and after treatment with melanoma cells. In this experiment the firefly luciferase gene was introduced into the melanoma cells (B16-luc2), and chemiluminescence was monitored. Tumour cell chemiluminescence was lower in the CXCL14 Tg mice than in the Wt animals, and the intensity of this chemiluminescence was increased in both Wt and Tg mice following injection of anti-NK1.1 (Fig. 3g). Further, we observed a correlation between the intensity of the chemiluminescence and the number of surface tumour metastases on the lungs (Fig. 3g and h), indicating that the number of surface metastases is likely reflected the number of tumour cells present throughout the lung (See Supplementary Figure S1).

Using this correlation, we observed a significant difference in the rate of lung metastasis between Wt and Tg animals (Fig. 3h, *P* < 0.001) prior to anti-NK1.1 injection, followed by a significant increase in the number of metastatic nodules in both Wt and Tg mice (Fig. 3h, *P* < 0.001) by the injection of the antibody. Notably, the effects of the antibody injection were more pronounced in the Tg mice, with the number of metastatic nodules increasing 25-fold, while only increasing 7-fold in the Wt animals, and thus decreasing the Wt/Tg ratio (R) from 5 to 1.5 (Fig. 3h).

In order to examine the effects of CXCL14 expression in the tumour cells on the rate of metastasis, we produced B16 melanoma cells that expressed the CXCL14 genes under the control of doxycycline (Dox). When the B16 melanoma cells (B16-luc2Tet/OnBRAK) were injected into Wt C57BL/6 mice, the number of metastatic nodules on the lungs was significantly lower in the Dox-treated mice (Fig. 3j, C57BL/6). When the cells were injected into T-, NKT-, and B-cell-deficient SCID mice, the number of the nodules increased but still the number was lower in the Dox treated mice (Fig. 3j, SCID). On the other hand when the cells were injected into T-, NKT-, B- and NK-cell deficient NOG mice, the number of nodules increased 10 times compared with that in the Wt C57BL/6 mice; and the numbers between Dox treated and untreated mice were not different (Fig. 3j, NOG).

### CXCL14 and α-galactosylceramide synergy during the suppression of B16-luc2 cell metastasis

**Figure 4:**
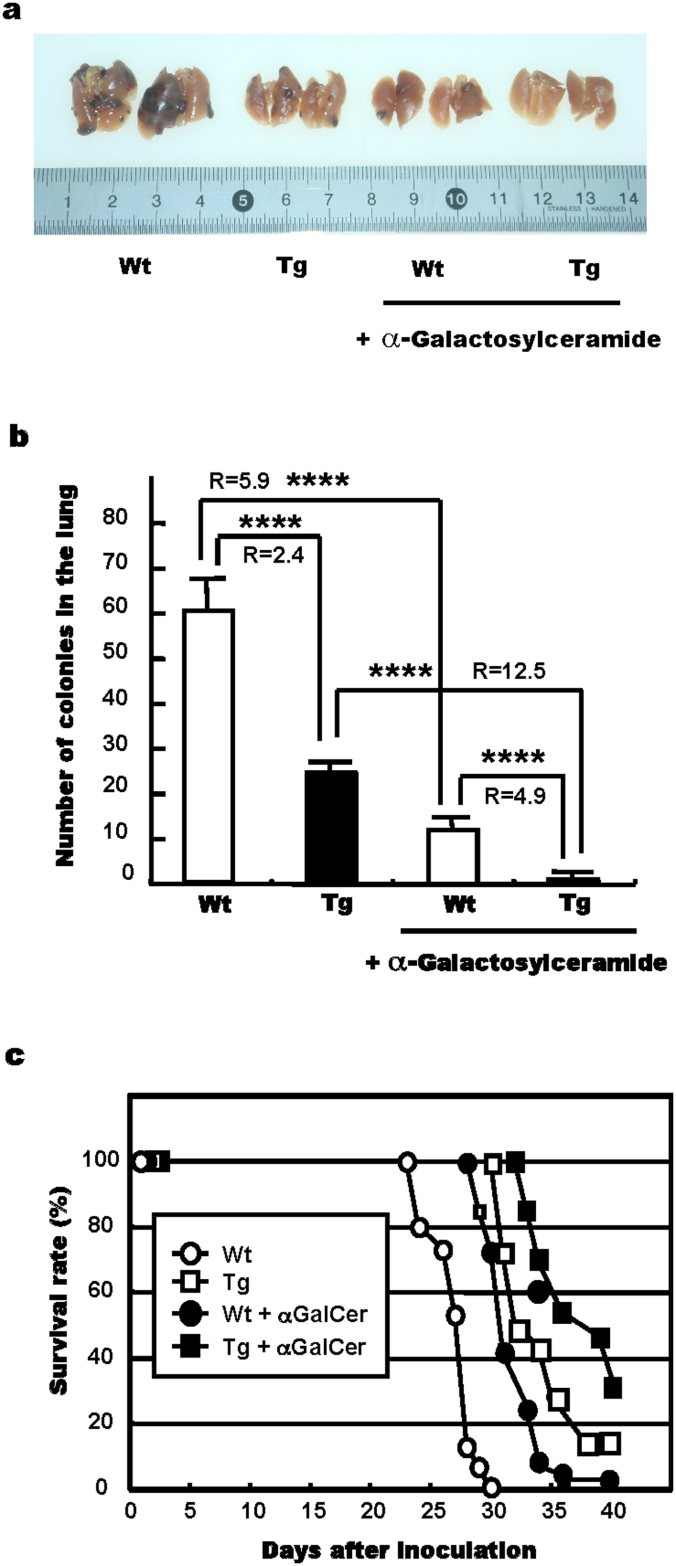
Synergistic effects of CXCL14 and α-galactosylceramide on the suppression of B16-luc2 LMT-3 cell metastasis. B16-luc2 LMT-3 melanoma cells (1 × 10^5^ cells/200 μL PBS), expressing the luciferase reporter gene, were injected into a tail vein of 12 each of the Wt and Tg mice. Half of the mice were injected with α-galactosylceramide (αGalCer, 2 μg/100 μL PBS/animal) 2 days before inoculation and twice a week thereafter to stimulate NKT cells. (a, b) The lungs were imaged (a) and the numbers of melanoma metastases were counted (b) at 4 weeks after inoculation of the melanoma cells. **** *P* < 0.0001, Student's *t*-test. (c) Survival rate following inoculation of the melanoma cells. Cells (2 × 10^5^ cells/200 μL PBS) were injected into a tail veins of 13 and 33 Wt and Tg mice, respectively; and α-galactosylceramide was injected into about half of these animals as described above. Wt (open circles) versus Tg (closed circles), *P* < 0.0001; Wt + α-galactosylceramide (open squares) versus Tg + α-galactosylceramide (closed squares), *P* < 0.05, Generalized Wilcoxon test. R indicates the ratios of metastatic nodule numbers between Wt and Tg mice or between mice pre-treated or not with α-galactosylceramide (connected with a bracket).

The lungs of B16-luc2 cell transplanted Wt and Tg mice were imaged 4 weeks after the melanoma cell injection with and without co-treatment with α-galactosylceramide, an NKT cell ligand and stimulator of NKT cell activity (Fig. 4a). The injection of α-galactosylceramide appeared to decrease the degree of pulmonary metastasis of B16-luc2 cells in both the Wt and Tg mice, but the effect was much stronger in the Tg mice (12.5-fold decrease) than in the Wt mice (5.9-fold decrease; Fig. 4b), indicating that the presence of both CXCL14 and α-galactosylceramide resulted in a synergistic effect and even greater tumour suppression. Similar synergistic effects were also observed in regards to the survival rate of the Wt and Tg mice (Fig. 4c), whereby the addition of α-galactosylceramide increased the life span of both the Wt and Tg mice, with the treated Tg mice living the longest.

### Increase in survival rate after injection of melanoma cells into CXCL14 transgenic mice

**Figure 5:**
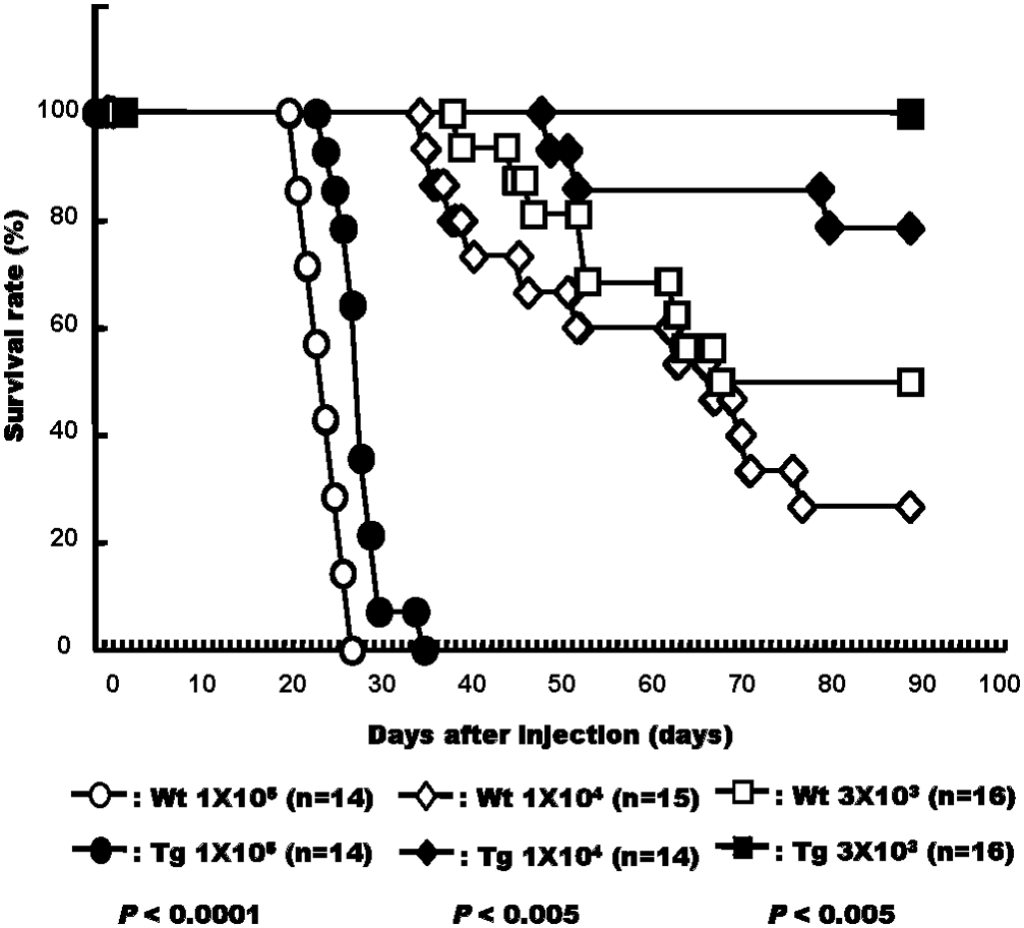
Survival rate of wild type (Wt) and CXCL14 transgenic (Tg) mice after injection of B16-luc2 LMT-3 melanoma cells. Variable concentrations of B16-luc2 LMT-3 cells (3 × 10^3^, 1 × 10^4^, or 1 × 10^5^ cells/200 μL PBS) were injected into a tail vein of Wt and Tg mice and the survival rate was determined at the indicated days following melanoma cell inoculation. The generalized Wilcoxon test was utilized.

In order to investigate effect of the higher expression of the *CXCL14* gene in the mice on the life span of the animals, we used the Kaplan-Meier method to determine the survival rates after the injection of various numbers of B16-luc2 cells (Fig. 5). The rate of survival was always significantly higher, *P* < 0.005 (3 × 10^3^ cells), *P* < 0.005 (1 × 10^4^ cells), *P* < 0.0001 (1 × 10^5^ cells), in the Tg mice than in the Wt mice, indicating that high expression of CXCL14 increased the survival rate as well as decreased tumour cell metastasis.

## Discussion

In order to investigate the effect of CXCL14 overexpression on the processes of carcinogenesis, increase in tumour volume, and metastasis, we utilized three lines of Tg mice, all of which ubiquitously express approximately 10-fold more CXCL14 than normal Wt mice^21^ (Also refer to Animals under Method section). Notably, the average level of CXCL14 in the blood plasma of Wt mice is comparable to that in humans, irrespective of the presence of tumour transplants, being approximately 0.9 ng/mL^21^,^25^. By using the AOM/DSS system, we found a significant decrease in the numbers of carcinoma formed in Tg mice compared with those in the Wt mice (Fig. 1e). The numbers of carcinomas formed in Wt and Tg mice were lower than those reported earlier for BALB/c mice, and so this difference may be strain dependent as reported^26^. AOM/DSS treatment of mice affects various functions of cells including stimulation of LGR5 positive stem-like cells^27^. Presently we detected a significant increase in the relative abundance of NKT cells (Fig. 1f) and suppression of STAT3 activation in Tg mice (Fig. 1g). NKT cells secrete interferon-γ, which induces tumour cell apoptosis and also stimulates NK cell maturation^28^,^29^. Activation of STAT3 suppresses apoptosis of cells. Our data obtained here suggest that expression of CXCL14 would have stimulated apoptosis of tumour cells^20^ to a greater extent in Tg mice than in Wt mice and support the data showing a decrease in the carcinomas in Tg mice compared with that in Wt ones.

Further, these CXCL14-overexpressing mice were subsequently injected with B16 melanoma or LLC cells in order to show the effects of high-level CXCL14 expression on the tumour growth and metastasis of these cell types. Importantly, these cell lines were chosen in part because they do not produce their own CXCL14; and so any effects could be expected to be a result of the overexpressed transgene in the microenvironment. In fact, we observed that the number of tumours that developed from the cells transplanted into the Tg mice was significantly lower than that found when the cells were injected into the Wt mice (Fig. 1). This observation not only confirmed previous data^21^, but also indicated that the size of the tumour developed from endogenous or transplanted cells was in large part suppressed by the environmental presence of CXCL14. Moreover, we observed significant increases in the tumour size and metastatic rate for both the Tg and Wt mice following treatment with anti-asialo-GM1 antibody, which act to deplete the NK cells, indicating that NK cell activity was important for the suppression of tumour growth and metastasis in both Wt and CXCL14 Tg mice (Figs. 2 and 3). We also produced CXCL14-expressing B16 melanoma cells under the control of Dox (B16-luc2Tet/OnBRAK). When the cells were injected into Wt and SCID mice treated with or without Dox in the drinking water, we found a decrease in the number of metastatic nodules in the lungs of mice treated with Dox, but none in the NK-cell deficient NOG mice (Fig. 3j), These data also support our contention that NK cells played an important role in the suppression of melanoma cell metastasis by CXCL14. It is reported that CXCL14 stimulates the migration of activated NK cells^30^. However, stimulation of only migration cannot explain the increase in cytotoxic activity observed here, because in CXCL14 Tg mice the levels of CXCL14 would be expected to be ubiquitously high.

When we isolated NK cells from the lungs of both Wt and Tg mice and compared their cytolytic activities against YAC-1 cells and B16 melanoma cells, but we could not detect significant differences in the activities of NK cells obtained from Wt and Tg mice (Supplemental Fig. S2). Nevertheless, the possibility remains that a small difference that could not be detected by the method employed here and/or mechanisms regulating NK cell activity by CXCL14 only *in vivo* could have caused the difference observed between Wt and Tg mice.

The rate of tumour growth following the addition of the NK-cell depleting antibody was higher in the Tg mice than in the Wt ones, but even so the final size of the tumour was smaller in the Tg mice.

NKT cells are also known to suppress tumour cell metastasis^31^ and activated NKT cells produce interferon γ, which in turn stimulates the activity of NK cells^28^,^29^,^32^. Therefore, it seemed likely to us that NKT cell activation, achieved with α-galactosylceramide, a ligand and activator of NKT cell receptors, would also affect the metastasis of melanoma cells in Tg and Wt mice. To test this possibility, we injected α-galactosylceramide into both groups of animals prior to melanoma cell transplantation. We observed an added level of metastasis suppression, with the relative rates of suppression being higher for the CXCL14 Tg mice than for the Wt ones. These data indicate a synergistic effect between CXCL14 and α-galactosylceramide. Furthermore, this synergism was also observed in terms of the increase in the survival rates for both the Tg and Wt mice, although it should be noted that the overexpression of CXCL14 alone increased the survival rate to some extent compared with the rate for the Wt ones, even when variable amounts of tumour cells were injected (Fig. 5). These data suggest that CXCL14 stimulated NK cell activity by targeting the point different from that of interferon-γ.

The data obtained here also suggest that CXCL14-suppressed tumour growth was not solely regulated by NK cells and that other factors, such as inhibition of tumour angiogenesis^21^,^33^ and tissue settlement rate of tumor cells^34^, also likely played roles in this suppressive process.

Notably, angiogenesis inhibitors are clinically employed as anti-cancer drugs as they have been shown to inhibit the growth of primary tumours, but this treatment does not increase the survival rates of patients, as these drugs often seem to simultaneously stimulate tumour cell invasion and metastasis^35^,^36^,^37^. In the CXCL14 transgenic mice described here, tumour metastasis and tumour volume were suppressed, and the survival rate was increased, results are quite different from those described above for angiogenic inhibitors.

Importantly, no specific receptor for CXCL14 has been identified. Recently, it was reported that CXCL14 competitively binds to chemokine receptor CXCR4, the specific receptor for CXCL12^38^,^39^, and inhibits its action. This is very interesting because the CXCL12-CXCR4 axis plays a pivotal role in the stimulation of tumour growth and metastasis^40^.

Thus, it seems plausible that inhibition of CXCR4 receptor activity in the tumour cells would have resulted in the suppressed growth and metastasis we observed in this study. In fact, AMD3100, a specific CXCR4 antagonist, effectively reduced tumour growth and ascites formation in a nude mouse model^41^. Furthermore, following liver injury, increased CXCR7, another CXCL12 receptor, stimulates regeneration, but suppression of CXCR7 function stimulates CXCR4 and induces liver fibrosis instead of regeneration^42^. These data suggest that CXCL14 may also inhibit tumour growth and metastasis by binding to CXCR4 and inhibiting CXCL12 activity. Unfortunately, without additional knowledge concerning the specific CXCL14 receptor, the molecular mechanism responsible for the stimulation of NK cell activity by CXCL14 *in vivo* remains unknown.

It has been reported that overexpression of CXCL14 in mice exacerbates collagen-induced experimental arthritis^43^, but that this exacerbation is largely dependent on the specific genetic background of the mouse^44^. And this arthritis model is only induced by injecting chicken collagen emulsified in Complete Freund's adjuvant. In fact without injection of the mixture, they could not find any alteration in the numbers of lymphocytes, dendritic cells, and macrophages in bone marrow, thymus, spleen, and lymph nodes in their unmanipulated Tg mice^43^. The risk of inducing arthritis was very low in our Tg mice. In fact, these animals showed no histologic abnormalities up to 2 years of age^21^, and the birth rate of our Tg mice was the same as that for the Wt mice (Supplemental Table S1). Interestingly, in a normal human population 2% of the individuals examined expressed blood levels of CXCL14 that were significantly higher than the average. In order to examine the reproducibility of the value obtained, we recollected a blood sample from 7 subjects at 3 months and from 6 subjects at 6 months after the first examination and determined their blood CXCL14 levels. The values obtained were constant regardless of the time of blood collection. One individual who constantly expressed blood CXCL14 levels during the 6-month chase period and that were nine times higher (8.3 ~ 8.5 ng/mL) than the average level and much closer to the levels observed in our Tg mice, and yet this individual showed no apparent abnormalities^25^. These findings also support the possibility that CXCL14 expressed at a high level would not cause severe side effects.

CXCL14 is expressed ubiquitously and constitutively in epithelia throughout the body^45^, and there are apparently contradictory data in the literature regarding the relationship between CXCL14 expression and tumour formation. For example, down-regulation of CXCL14 expression has been associated with multiple adenocarcinomas, such as those of the prostate^46^ and lungs^47^, as well as colon carcinomas^48^ and HNSCC^17^,^18^,^19^,^20^. On the other hand, in some other reports heightened expression of this chemokine was observed in these same types of carcinomas and adenocarcinomas^49^,^50^,^51^. Recently, the production of CXCL14 by cancer cell associated fibroblasts was also suggested^52^, indicating an additional mechanism by which this chemokine could possibly regulate tumour growth. There are several possible explanations for the apparent discrepancy in the effects of CXCL14 on tumour progression, *e.g.*, cell type-specific functions and/or stage-specific effects of CXCL14 during tumour progression. It is also conceivable that CXCL14 may play an opposing role when combined with other factors or in the presence of modified molecules of CXCL14. Of the conceivable explanations for these discrepancies, our data exclude the possibility of any cell type-specific or stage-specific activity differences, suggesting that the opposing effects of CXCL14 likely occur because of the presence of other factors and/or modified molecules of CXCL14 that have different functions.

In conclusion, we demonstrated that CXCL14 Tg mice showed a suppressed rate of carcinogenesis, decreased tumour volume, and reduced pulmonary metastasis, as well as an increased survival rate of mice following tumour cell injection. There are two other transgenic mouse models for tumour suppression, one targeting Par-4^53^ and the other, PTEN^54^. Because these target genes encode intracellular proteins, the transduction efficiency in the cells would be major problem for their clinical application. CXCL14, on the other hand, can function in the microenvironment, resulting in similar levels of tumour suppression when forced to be expressed in the cells, without any observable side effects. Thus, we believe that our CXCL14 Tg mouse model may be useful for investigation of the molecular mechanisms involved in multi-step tumour progression and suppression and that further research concerning the clinical application of CXCL14 to treat cancer is warranted.

## Methods

### Animals

Three independent C57BL/6 mouse lines overexpressing the *CXCL14* gene, under the control of a beta-actin promoter and CMV enhancer were produced as described previously^21^. Homozygous line 20 (RBRC02382 C57BL/6J-Tg[CXCL14]-1) mice expressed 14.8 ± 1.3 ng/mL of plasma CXCL14 protein, whereas the heterozygous line expressed 6.6 ± 1.0 ng/mL, Lines 27 (RBRC02383 C57BL/6J-Tg[CXCL14]-2) and 52 (RBRC02384 C57BL/6J-Tg[CXCL14]-3) were heterologous for the CXCL14 gene and their plasma protein levels were 11.0 ± 1.1 ng/mL and 8.6 ± 0.9 ng/mL, respectively. Wt mice had only 0.9 ± 0.1 ng/mL of CXCL14 in their plasma. These three lines of CXCL14 Tg mice showed normal fertility rates and offspring viability (Supplementary Table S1).

Male and female mice, 8–14 weeks in age, from each of the above three animal lines were used for experiments. At least six mice (n = 6) were used per group for each experiment. All methods were performed in accordance with the protocols approved by The Institutional Animal Care and Use Committee of Kanagawa Dental University and that of Kanazawa University, respectively.

### Chronic colitis-associated colorectal cancer

Pathogen-free, 8- to 12-week-old C57BL/6 Wt (Charles River Laboratories, Yokohama, Japan) and CXCL14 Tg (heterologous line 20) were housed under specific pathogen-free conditions at the animal facilities of Kanazawa University. Mice were injected (*i.p.*) with 12-mg/kg- body weight of AOM (Sigma-Aldrich, Inc. St Louis, MO), a carcinogen that is widely used to induce tumours in the distal part of colon by O^6^-methylguanine formation. AOM was dissolved in physiologic saline. The mice were then given intermittent additions of 3% DSS (Mw 36K-50K, MP Biochemical Japan, Tokyo, Japan), known to cause an acute inflammatory reaction and ulceration in the entire colon similar to that observed in ulcerative colitis, in the drinking water, as shown in Fig. 1a. The animals were sacrificed at selected times for macroscopic inspection and histologic analysis. Resected mouse colon tissue was fixed in 10% formalin neutral buffered solution (Wako Pure Chemical Industries Ltd. Osaka Japan) prior to paraffin embedding. Sections were cut at 5 μm and stained by common histological techniques (e.g., HE staining). Paraffin-embedded sections were also deparaffinized for immunohistological detection of F40/80 (a macrophage marker) and Ly6G (a neutrophil marker), as described previously^55^.

### Preparation of cell fraction for FACS analysis

Colon tumour tissues were obtained from the mice at 56 days after the initiation of DSS intake and were opened longitudinally. Single cell suspension was prepared as described previously^56^. The resulting single-cell suspensions were incubated with the combination of FITC-conjugated hamster anti-mouse CD3 mAb (eBioscience, San Diego, CA) and PerCP-Cyanine5.5-conjugated mouse anti-mouse NK1.1 mAb (eBioscience) for 20 minutes on ice. Isotype-matched control immunoglobulins were used to detect nonspecific binding of immunoglobulin. The stained cells were analyzed on a FACSCanto System II (BD Bioscience) with gating on lymphocyte fractions based on forward and side scatter light intensities. CD3−, NK1.1+ gated cells were defined as NK cells and CD3+, NK1.1+ gated ones were defined as NKT cells.

### Western blotting analysis

For western blotting analysis, colon tissues were collected and homogenized with a RIPA lysis buffer (Santa Cruz Biotechnology, Santa Cruz, CA). After separation with SDS/polyacrylamide gel electrophoresis, proteins were transferred onto nylon membranes and then reacted with rabbit anti-mouse STAT3 and rabbit anti-mouse Phospho-STAT3 (Tyr705) antibodies (Cell Signaling Technology, Beverly, MA). Anti-β-actin antibody (Cell Signaling Technology) was used to confirm that equal amounts of proteins had been used for analysis. Signals were detected by using an LAS-4000 (GE Healthcare Japan, Hino, Japan).

### Immunohistochemical analyses of mouse colon tissues

Resected mouse colon tissues were fixed in Tissue-Tek Ufix (Sakura Fine Technical Co., Tokyo, Japan) for paraffin embedding. For antigen retrieval of paraffin sections, the deparaffinized slides were either autoclaved in 10 mmol/L citrate buffer (pH 6.0) for 20 min at 121°C. Endogenous peroxidase activity was blocked by using 0.3% H_2_O_2_ for 15 minutes, followed by incubation with Blocking One Histo (Nacalai Tesque, Tokyo, Japan) for 15 minutes. The sections were incubated with the anti NF-κB p65 antibody (Cell Signaling) overnight in a humidified box at 4°C. The resultant immune complexes were then detected by use of an ABC Elite kit (Vector Laboratories, Burlingame, CA) and peroxidase substrate 3,3′-diaminobenzidine kit (Vector Laboratories), according to the manufacturer's instructions. Representative results from 5 independent experiments are shown here.

### Tumour cell transplants and determination of tumour volume *in vivo*

C57BL/6 mouse derived LLC cells (RCB0558) and B16 melanoma cells (RCB1283) were obtained from the Riken Cell Bank and cultured as described previously^21^. Cells in the log phase were suspended in Dulbecco's phosphate buffered saline (DPBS(-); Wako Pure Chemical Industries, Osaka), and injected subcutaneously into both sides of the dorso-lateral region of 6 to 10 mice per experimental group. Tumour volume was then calculated according to the following formula^19^: (a × b^2^)/2, where a is the longer dimension and b is the smaller. The volumes calculated with this formula were closely related to the weight of the tumours isolated after sacrifice. In order to deplete NK cell activity, an anti-asialo-GM1 rabbit antibody (Wako Pure Chemical Industries, Osaka, 0.5 mg/200 μl DPBS (-)) was injected (*i.p.*) 3 days before the injection of the tumour cells and then once a week thereafter.

### Experimental metastasis and colonization to the lungs

LLC and B16 melanoma cells were cultured in Dulbecco's modified Eagle's medium containing antibiotics and 10% fetal bovine serum (FBS) as described above. Luciferase expressing B16 melanoma cells (B16-luc2; Caliper Life Sciences, Alameda CA) were cultured in RPMI-1640 medium (Life technologies, Tokyo) containing antibiotics and 10% FBS. The metastatic rate of the B16-luc2 cells was lower than that of the original B16-F10 cells; and thus in order to obtain clones of higher metastatic activity, the isolated cell colonies (B16-luc2) were allowed to metastasized three times in the lungs i*n vivo* (B16-luc2 LMT-3).

The cells were dispersed by trypsin treatment and then incubated for 1 hour at 37°C under 95% air and 5% CO_2 _in FBS containing medium to restore the cell surface damaged by the trypsin treatment. After recovery from the trypsin treatment, the cells were rinsed 3 times with DPBS (-) and then resuspended in it, and then injected into the tail vein of the mouse (200 μL of solution containing 3 × 10^3^ to 2 × 10^5^ tumour cells). The number of nodules of metastatic tumour cells in the lungs was determined by using a dissection microscope (Nikon, Tokyo) and the weight of the lungs was determined after fixing them with 10% formalin. In some experiments, anti-asialo-GM1 antibody (0.5 mg/200 μL DPBS (-)/animal) was injected (*i.p.*) 1 or 3 days before tumour cell injection and then once or thrice a week thereafter, in order to deplete NK cell activity. Injection of the antibody whether once or thrice a week did not affect the metastatic rate of the melanoma cells. In other mice, mouse monoclonal anti-NK1.1 antibody (50 μg/50 μL DPBS (-)/animal; BD Pharmingen, Tokyo) was also injected (*i.p.*) 3 days before melanoma cell inoculation and once a week thereafter to deplete NK and NKT cell activity.

### Stimulation of NKT cell activity

In order to stimulate NKT cell activity, half of the experimental groups of animals were injected (*i.p*.) with α-galactosylceramide (α-GalCer, KRN7000; Funakoshi Tokyo, Japan) 1 or 2 days before injection of tumour cells and once (20 μg) or twice (2 μg) per week thereafter.

### Determination of chemiluminescence

Luciferase activity of cultured B16-luc2 cells was determined as described previously^57^. Fifty cells expressing chemiluminescence corresponded to 1 pg of luciferase. The metastatic rate of the B16-luc2 cells was lower than that of the original B16-F10 cells, as explained above; so these cells were allowed to metastasize and be selected by repeated isolation of metastatic nodules to the lungs. Notably, cell colonies selected three times, designated B16-luc2 LMT-3, and expressed levels of luciferase activity comparable to those of the original B16-luc2 cells. *In vivo* chemiluminescence images were obtained by using an IVIS-50 (Caliper Life Sciences, Alameda CA) 30 minute after injection of the luciferin solution from both sides of the peritoneum (3 mg/100 μl DPBS (-); VivoGlo luciferin, Promega, Tokyo).

### Statistical analysis

The Student's *t*-test was used to assess statistically significant differences between two groups. The survival curves were plotted according to the Kaplan-Meier method and the statistical difference was checked by using the generalized Wilcoxon test. A value of *P* < 0.05 was considered statistically significant.

## Author Contributions

R.H., Y.K., N.M. and M.T. were responsible for the experimental design associated with this research. R.H., K.I., Y.K., S.S., Y.M., C.M., T.A., X.Y., K.T., Y.N. and T.K. performed the research described in this manuscript. R.H., K.I., Y.K., S.S. and Y.N. analyzed the data. Notably, R.H., K.I., Y.K. and S.S. contributed equally to this work. R.H., Y.N., N.M. and M.T. wrote the paper.

## Supplementary Material

Supplementary Information Supplementary Information
