# Supplementary material for: Suppressed rate of carcinogenesis and decreases in tumour volume and lung metastasis in CXCL14/BRAK transgenic mice
Source: Sci Rep. 2015 Mar 13;5:9083. doi: 10.1038/srep09083 (PMC4357995; doi:10.1038/srep09083)
Supplement: Supplementary Information [file srep09083-s1.pdf]

Supplementary Information

Suppressed rate of carcinogenesis and decreases in tumour volume and lung metastasis in CXCL14/BRAK transgenic mice

Ryu-Ichiro Hata[*,1,2], Kazuhito Izukuri[1,2], Yasumasa Kato[3], Soichiro Sasaki[4], Naofumi Mukaida[4], Yojiro Maehata[1,2], Chihiro Miyamoto[1,2], Tetsu Akasaka[1,2], Xiaoyan Yang[1,2], Yoji Nagashima[5], Kazuyoshi Takeda[6,**], Tohru Kiyono[7] & Masaru Taniguchi[8]

[1]Oral Health Science Research Center and [2]Department of Oral Science, Graduate School of Kanagawa Dental University, Yokosuka, 238-8580, Japan; [3]Department of Oral Function and Molecular Biology, Ohu University School of Dentistry, Koriyama, 963-8611, Japan; [4]Division of Molecular Bioregulation, Cancer Research Institute, Kanazawa University, Kanazawa, 920-1192, Japan; [5]Department of Molecular Pathology, Yokohama City University Graduate School of Medicine, Yokohama, 236-0004, and Department of Surgical Pathology, Tokyo Women's Medical University Hospital, Tokyo, 162-8666, Japan; [6]Department of Immunology, Juntendo University School of Medicine, Tokyo, 113-8421, Japan; [7]Division of Carcinogenesis and Cancer Prevention, National Cancer Center Research Institute, Tokyo, 104-0045, Japan; and [8]Laboratory for Immune Regulation, RIKEN Center for Integrative Medical Sciences, Yokohama, 230-0045, Japan.

*Correspondence and requests for materials should be addressed to Ryu-Ichiro Hata, Oral Health Science Research Center, Graduate School of Kanagawa Dental University, Yokosuka, 238-8580, Japan, Phone: +81-46-822-9587; Fax+81-46-822-9587; E-mail: hata@kdu.ac.jp

** Present address
Division of Cell Biology
Biomedical Research Center
Graduated School of Medicine
Juntendo University
Tokyo 113-8421, Japan

20 line			
157			
male		female	
82 (53.2%)		75 (47.8%)	
Wt	Tg	Wt	Tg
36 (22.9%)	46 (29.3%)	36 (22.9%)	39 (24.8%)

27 line			
370			
male		female	
174 (47.0%)		196 (53.0%)	
Wt	Tg	Wt	Tg
89 (24.1%)	85 (23.0%)	102 (27.6%)	94 (25.4%)

52 line			
534			
male		female	
269 (50.4%)		265 (49.6%)	
Wt	Tg	Wt	Tg
141 (26.4%)	128 (24.0%)	140 (26.2%)	125 (23.4%)

Table S1. All three lines of CXCL14 transgenic mice show normal fertility and viability

Note: The three CXCL14 transgenic (Tg) founders were crossed with isogenic wild type (Wt) C57BL/6 mice and the birth rates of male and female were determined for each line. There were no significant differences between the distribution of sex and Tg genes. Line 20 (RBRC02382 C57BL/6J-Tg[CXCL14]-1), line 27 (RBRC02383 C57BL/6J-Tg[CXCL14]-2), and line 52
(RBRC02384 C57BL/6J-Tg[CXCL14]-3) were employed in this study.

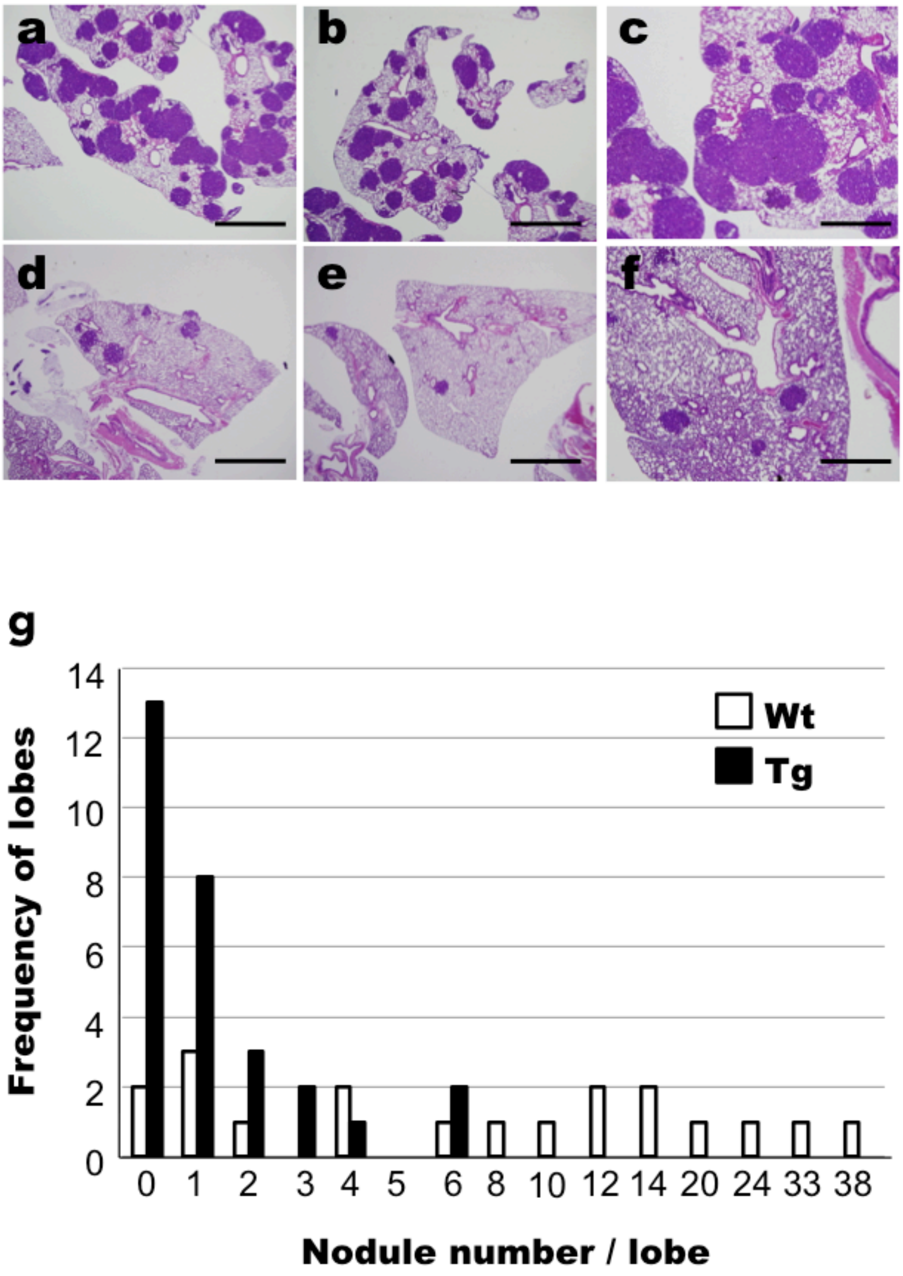
a
b
c
d
e
f
g
Wt
Tg
Frequency of lobes
0
2
4
6
8
10
12
14
0 1 2 3 4 5 6 8 10 12 14 20 24 33 38
Nodule number / lobe

Figure S1. Comparison of metastatic nodules in lungs of wild type (Wt) and Tg mouse

Eighteen days following injection of B16 melanoma cells into a tail vein, the lungs were sampled, fixed in 10% formalin, and embedded in paraffin for histopathological examination. Sections (4 μm) were stained with H& E, and the number of metastatic nodules was counted in each lobe under a light microscope. The number of metastases correlated positively with the number of surface metastases observed with a dissection microscope, and thus we routinely used the number of surface metastases for comparison. Light micrographs of lungs obtained from the wild-type (a-c) and transgenic (d-f) mice after intravenous injection of melanoma cells and distribution of numbers of metastatic nodules per lobe (g).

Bars represent 1 mm (a, b, d, and e) or 0.5 mm (c and f).

Note the decreased numbers of metastatic nodules in the transgenic mice, compared with the number in the wild type.

(g) Frequency of lobes containing respective number of nodules was significantly different between those of Wt and Tg mice. $P<0.02$, Welch's *t*-test

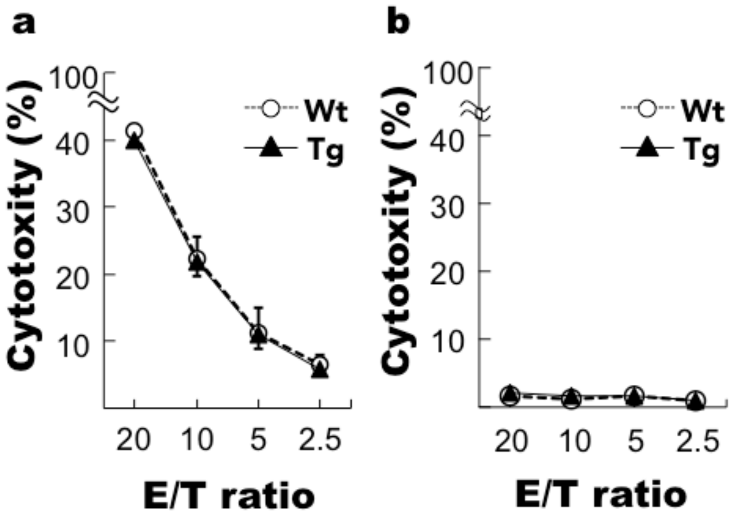

Figure S2. . Cytotoxic activities of NK cells obtained from lungs of Wt and Tg mice.

We isolated NK cells from the lungs of Wt (open circles) and Tg (closed triangles) mice by negative selection by using a mouse NK cell isolation kit (Milteny Biotec, Bergisch Glabach, Germany) as described previously[32]. Purities of NK cells were 76.7 % (Wt) and 75.8 % (Tg), respectively.

Immediately after purification of the NK cells from the lungs, their cytotoxicity was examined against YAC-1 (a) or B16 melanoma cells (b) at the indicated effector cell (E)/target cell (T) ratios, as described previously[32]. Data are presented as the means ± S.D. Representative data from triplicate experiments are shown.
